# Cost effectiveness of ulcerative colitis treatment in Germany: a comparison of two oral formulations of mesalazine

**DOI:** 10.1186/1472-6963-11-157

**Published:** 2011-07-05

**Authors:** Anne Prenzler, Linnette Yen, Thomas Mittendorf, J-Matthias von der Schulenburg

**Affiliations:** 1 Leibniz University Hannover, Center for Health Economics, Hannover, Germany; 2 Global Health Economics and Outcomes Research Shire Pharmaceuticals, Wayne, Pennsylvania, USA; 3 Herescon GmbH-Health Economic Research & Consulting, Hannover, Germany

**Keywords:** Ulcerative colitis, mesalazines, cost effectiveness, cost analysis, Mezavant, Asacol

## Abstract

**Background:**

The treatment of ulcerative colitis (UC) can place a substantial financial burden on healthcare systems. The anti-inflammatory compound 5-aminosalicylic acid (5-ASA; mesalazine) is the recommended first-line treatment for patients with UC. In this analysis, the incremental cost effectiveness ratio (ICER) of two oral formulations of 5-ASA (Mezavant^® ^and Asacol^®^) is examined in the treatment of patients with mild-to-moderate, active UC in Germany.

**Methods:**

A Markov cohort model was developed to assess the cost effectiveness of Mezavant compared with Asacol over a 5-year period in the German Statutory Health Insurance (SHI). Drug pricing details for 2009 were applied throughout the model, and overall resource use was determined and also fitted to 2009 from published results of a large cross sectional study of German SHI patients. Cost per quality adjusted life year (QALY) was the primary endpoint for this study. Remission rates were obtained using data from a randomised, phase III trial of Mezavant with an active Asacol reference arm and a long-term, open label, safety and tolerability trial of Mezavant. Uncertainty in the study model was assessed using one-way and probabilistic sensitivity analyses applying a Monte Carlo simulation.

**Results:**

Over a 5-year period, healthcare costs for patients receiving Mezavant were 624 Euro lower than for patients receiving Asacol. Additionally, patients receiving Mezavant gained 0.011 QALYs or 18 more days in remission compared with Asacol. One-way sensitivity analyses suggest that these results are driven by both differences in the acquisition cost between mesalazine formulations and differences in treatment efficacy. Furthermore, sensitivity analyses suggest a probability of 76% for cost savings and higher QALYs with Mezavant compared with Asacol. If adherence and its influence on the remission rates and the risk of developing colorectal cancer were included in the model, the results might have even been more favorable to Mezavant due to its once daily dosing regimen.

**Conclusions:**

This model suggests that patients treated with Mezavant may achieve increased time in remission and higher QALYs, with lower direct costs to the SHI when compared with Asacol. Mezavant may therefore be a suitable first-line option for the induction and maintenance of remission in UC.

## Background

Ulcerative colitis (UC) is a relapsing non-transmural inflammatory bowel disease that is restricted to the colon. Patients typically have bloody diarrhea (often nocturnal and postprandial), passage of pus, mucus, or both, and abdominal cramping during bowel movements [1]. This chronic symptomatic morbidity is associated with impairment of health-related quality of life, higher unemployment, and productivity loss [2].

In Germany, there are about 160,000 prevalent UC-cases [3]. The incidence of UC amounts to around 6/100,000 cases a year [4-6]. The treatment of these patients during the different disease states can place a substantial financial burden on the healthcare system. For example, the German Federal Statistical Office estimated health care costs due to UC at 233 million Euro in 2006 [7]. Hence, analyses which compare cost and benefit of different health care interventions become more and more important for the German health care sector in order to identify cost effective treatment options.

In Germany, several medications are approved for the treatment of UC-patients during remission and active states. Today, the recommended first-line treatments for mild to moderate UC are anti-inflammatory 5-aminosalicylic acid (5-ASA) preparations containing sulfasalazine, mesalazine or olsalazine. Since February 2008, a new mesalazine preparation (Mezavant^®^, Shire Pharmaceuticals Inc.)-utilizing MMX^® ^technology (trademark of Cosmo Technologies Ltd, Wicklow, Ireland)-is available in Germany. Mezavant is indicated for the induction of clinical and endoscopic remission in patients with mild-to-moderate, active UC as well as for the maintenance of remission. The delivery system of Mezavant uses lipophilic and hydrophilic matrices enclosed within a gastro-resistant, pH-dependent coating in order to facilitate prolonged exposure of the colonic mucosa to mesalazine. Mezavant is administered only once a day with tablets containing 1,200 mg of mesalazine; this differs from some other oral mesalazines, e.g. Asacol^®^, which is available in 400 mg tablets and administered two or three times daily. Asacol (Procter & Gamble, USA) is also indicated for mild-to-moderate acute exacerbations of UC and the maintenance of remission of UC. The simple dosing regimen of Mezavant with only once daily intake of 2 to 4 tablets may have potential benefits in comparison to other treatment options by improving patient adherence, which in turn may affect the recurrence of disease exacerbations and reduce resource utilization such as surgery and hospitalization and associated costs.

However, as stated above, the cost effectiveness of new treatment options becomes more and more important in the German setting. Therefore, the aim of this study is to examine the cost effectiveness of Mezavant in comparison to Asacol in the treatment for induction and maintenance of remission in patients with mild-to-moderate UC in Germany.

## Methods

To assess the cost effectiveness of Mezavant compared with Asacol, a Markov cohort model was developed. The primary endpoint in this analysis was incremental cost per additional quality-adjusted life year (QALY) and the evaluation was conducted from the perspective of the Statutory Health Insurance (SHI) in Germany. The structure of the model, the input data (transitions probabilities, utilities and costs) as well as the analysis procedure is described in detail below.

### Structure of the markov model

The model [8] simulates the dynamic treatment path of an UC-patient. The modeled treatment period in the base case was five years, divided into 8-week cycles which match the reporting period of the clinical trials [9,10]. In total, the model includes eight possible health states.

**Figure 1:**
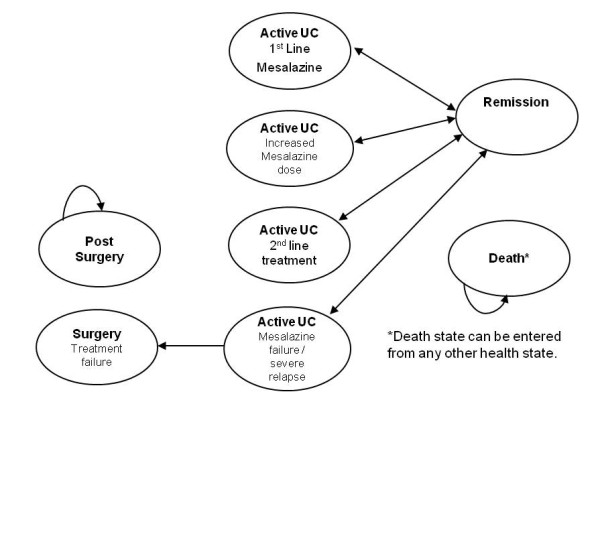
**Structural diagram of the cost effectiveness model**.

In first line treatment, newly diagnosed or relapsing patients (relapsed ≤ 6 weeks prior to baseline) with active, mild-moderate UC receive either Mezavant of Asacol, each at 2,400 mg a day. Patients who do not respond to the first line treatment receive an increased dosage (4,800 mg/day) of Mezavant or Asacol, respectively. In case the increased dosage does not lead to a remission, oral corticosteriods are added to the mesalazine treatment (second line treatment). In case the second line therapy also fails, it is assumed that patients need hospitalization (without surgery) to receive immunosuppressants and/or intravenous steroids [8]. Hospitalization including surgery is necessary if the treatment still does not lead to a remission for patients. Patients, who have undergone surgery, enter the post-surgery state. It is assumed that these patients are not in an active state anymore. Remission is defined as a status in which patients have no or reduced UC symptoms, which also might be entered from all health states except surgery, post-surgery and death. The death state can be entered from any status either due to all other cause mortality or surgery. The structure of the cost effectiveness model is presented in Figure 1.

### Transition probabilities

Remission rates were obtained using data from two clinical trials: one phase III, randomized, placebo-controlled study [9] and one open-label extension study [10] which assessed the long-term safety, tolerability and maintenance of remission. The efficacy of Mezavant was also assessed in another clinical trial (Lichtenstein et al. (2007) [11]). However, since this trial did not include an active comparator reference arm, the results of the study were not eligible for this kind of comparing cost effectiveness analysis.

Within the phase III study [9], UC-patients were treated with a once daily active dose of 2,400 mg Mezavant, 2,400 mg Asacol (divided into three doses of 800 mg each) or placebo (administered as double-dummy) for eight weeks. (Furthermore, in one treatment arm patients were initiated on treatment with a higher dose of Mezavant (4,800 mg) in the trial; however, the results of this arm were not included in the model, since no comparable dosing regimen for Asacol is available.) The remission rates within this time period were 40.5% for patients treated with 2,400 mg Mezavant, 32.6% for patients treated with Asacol and 22.1% for placebo. Remission was defined as a modified UC-DAI-score [made up of scores for rectal bleeding, stool frequency, physician global assessment, and sigmoidoscopic mucosal appearance] of ≤ 1 with a score of 0 for rectal bleeding and stool frequency as well as at least a 1-point reduction from baseline in the sigmoidoscopy score and no friability of the mucosa [9]. Patients (Asacol-and Mezavant-patients) who did not achieve remission during the eight weeks were enrolled in the open-label extension study [10] and received an increased dose of 4,800 mg/day Mezavant. Patients who had initially received Mezavant in the phase III trial achieved a remission rate of 61.5% in the open-label extension; Asacol-patients from the original pivotal trial, who were treated with an increased dose of Mezavant in the open-label extension study, achieved a remission rate of 61.0%. No trial data was available giving information on the remission rate of Asacol-patients who received an increased dose of Asacol. Therefore, the authors made a conservative assumption against Mezavant and supposed that the remission rate of Asacol-patients receiving an increased dose of Asacol is equal to the remission of Asacol-patients who received an increased dosage of Mezavant in the extension study (= 61.0%).

Besides the information from these two trials, further remission rates and transition probabilities were necessary for the setup of the cost effectiveness model. Remission rates from second-line treatment with corticosteroids (prednisolone) were reported by Lennard-Jones et al. with 68% [12]. As stated above, in the model it is assumed that those patients who do not achieve remission in second line treatment require hospitalization (health state: "Active UC: mesalazine failure"). The transition probability to the health state "Surgery" was taken from the official hospital statistics of the German Federal Statistical Office (10.9% of all hospitalized UC-patients require surgery) [13].

Furthermore, to determine an eligible probability of remaining in the remission state, the results of the studies of Prantera et al. [14] as well as Langholz et al. [15] were combined. Prantera et al. conducted a clinical study in Italy, Poland and Ukraine by comparing the maintenance of UC-patients in remission taking either Mezavant or Asacol over a period of one year. According to the results, after 12 months, 62.2% of the Mezavant-patients and 51.5% of the Asacol-patients (p < 0.05) were still in remission (based on the UC-DAI and patient diaries which document a worsening in stool frequency and increased rectal bleeding). If only the UC-DAI data was taken into account (without patient diary), the remission rates were 68.0 and 65.9%, respectively (p = 0.69). For the base case, however, the above mentioned rates which include information from patient diaries are applied, since regulatory guidelines stress the importance of obtaining direct patient input [e.g. [16]]. However, the other remission rates are applied in a sensitivity analysis. Within the model, the figures (62.2 and 51.5%) were then extrapolated to the base case study period of five years by applying findings from Langholz et al. [15]. The authors found out that the probability of experiencing a relapse diminishes by 1% each additional year spent in remission.

Age-specific overall cause mortality data (2005/2007) was taken from the German Federal Statistical Office [17]. Patients in the model started with an average age of 43.2 years and thus reflecting the underlying data from the clinical trial [9]. For patients, who undergo surgery, it was anticipated, that they have a higher mortality rate. However, for Germany no sufficient data regarding the higher mortality rate of UC-patients due to surgery could be identified, which led to the inclusion of UK-specific data. The UK Inflammatory Bowel Disease Audit [18] reports that of all UC inpatient admissions (2,767 inpatient admissions) 45 patients died. Of those, 25 deaths were directly related to UC including 15 that occurred in patients that underwent surgery (715 surgeries). This results in a mortality rate of 2.1% (15/715) directly due to surgery.

An overview and summary of the presented transition probabilities is given in table 1.

**Table 1:** Overview over the transition probabilities

	To ... (M: Mezavant cohort; A: Asacol cohort)							
**From ...**	**1^st ^line**	**1^st ^line increased dosage**	**2nd line**	**Failure/relapse**	**Surgery**	**Post surgery**	**Remission**	**Death**

Active UC: 1^st ^line		M: 59.5% A: 67.4%					M: 40.5% A: 32.6%	0.0%**

Active UC: 1^st ^line increased dosage			M: 38.5% A: 39.0%				M: 61.5% A: 60.0%	0.0%**

Active UC: 2nd line				M: 32.0% A: 32.0%			M: 68.0% A: 68.0%	0.0%**

Active UC: Failure/relapse					M: 25.8% A: 25.8%		M: 74.2% A: 74.2%	0.0%**

Surgery						M: 97.9% A: 97.9%		M: 2.1% A: 2.1%

Post surgery						M: 100.0% A: 100.0%		0.0%**

Remission	M: 6.5%* A: 9.2%*						M: 93.5% A: 90.8%	0.0%**

Death								M: 100.0% A: 100.0%

* This figure decreases as time spent in remission decreases the risk of active episodes [14,15]

** The overall cause mortality data [16] (dependent on the age cohort) is below 0.05% and is therefore displayed in the table as 0.0%.

### Utility data

Utility data for UC-patients was derived from two studies [19,20] in which utility scores were obtained via a Time Trade Off approach and the EQ-5D from a sample of 151 patients with UC. Simultaneously, the researchers also collected data on the clinical severity of the disease. The results of these studies were adapted to fit the specified model health states (see table 2)

**Table 2:** Utility values for modeled health states [19,20]

Health State	Utility (SD)	Disease severity/Assumptions
Active UC: 1^st ^line	0.589 (0.269)	Mild-to-moderate active disease (SCAI = 4-10)
Active UC: 1^st ^line increased dosage	0.589 (0.269)	Mild-to-moderate active disease (SCAI = 4-10)
Active UC: 2^nd ^line	0.589 (0.269)	Mild-to-moderate active disease (SCAI = 4-10)
Active UC: Failure/relapse	0.317 (0.315)	Severely active disease (SCAI > 10)
Surgery	0.317 (0.315)	Assumption: utility is equal to the utility of patients with relapse
Post surgery	0.845 (0.195)	Assumption: utility is equal to the utility of patients in remission
Remission	0.845 (0.195)	Patients in remission (SCAI < 4)

### Resource use and cost data

Resource use and costs were determined from published results of a large cross sectional study of patients with UC in Germany [21]. Within this study, 519 patients with UC from 24 ambulatory gastroenterological specialists' practices and two hospitals were enrolled. For these patients, outpatient and inpatient visits, outpatient procedures as well as prescribed medications were recorded. The resource use was evaluated from the perspective of the Statutory Health Insurance (SHI) in Germany, taking also into consideration patient co-payments as well as discounts given by the manufacturer and pharmacies as required by legal obligations in Germany. For this, the current German guideline [22] for the identification and valuation of resource usage was applied to evaluate the costs from the perspective of the SHI. The price year which was used in the UC-study [21] was 2007. For this present cost effectiveness analysis, the resource use collected in the cross sectional study was re-fitted to 2009 from the perspective of the German SHI on the basis of the current reimbursement practice. In the following, costs are described in detail:

#### Medication costs

According to the German guideline [22], the prices for medications are generally based on the largest available pack size. Furthermore, since prices of medications vary throughout a year, the costs from the middle of the year (July 1^st, ^2009) should be used for health economic evaluations. In Germany, mesalazine is part of the reference price system (Festbetrag) which defines costs per mg depending on pack size. In addition, since costs have to be valued from the perspective of the SHI, co-payments as well as manufacturer and pharmacy discounts need to be subtracted from the reference price. Taking all these requirements into consideration, the costs for a Mezavant (1,200 mg) tablet were 1.13 Euro and the costs for an Asacol (400 mg) tablet are 0.44 Euro. Costs for an oral corticosteroid (prednisolone), which is applied daily in the 2^nd ^line treatment in addition to mesalazine, are 0.25 Euro per 40 mg [23]. Since clinical guidelines suggest to reduce the dosage gradually within the 8-week-cycle, the prednisolone dose is reduced each week by 5 mg (after starting at 40 mg). Accordingly, the costs are reduced during the cycle.

#### Outpatient costs

In Germany (since 2009) physicians are reimbursed via lump sum payments per quarter, independently from the number of visits of a patient per quarter. According to the official German Uniform Valuation Scheme (EBM), gastroenterologists received 18.90 Euro per patient per quarter (not including special procedures) in 2009. It is assumed that this lump sum payment occurs once a quarter for patients in an active disease state. In the present model, the cycle length with eight weeks is less than a quarter. To follow a conservative approach, these costs were fit to the cycle length (18.90 · 8/13 = 11.63 Euro). Furthermore, the above mentioned German UC-study [21] documented the type and amount of outpatient procedures for active UC-patients (e.g. colonoscopy, ultrasonography). This resource use was revalued on the basis of the price year 2009. According to the calculations, a UC-patient in an active disease status caused outpatient procedures costs of 115.63 Euro within a six month period. To fit this data to the cycle length of eight weeks, these costs were divided by 26 and multiplied with eight weeks (115.63 · 8/26 = 35.58 Euro). In total, during the period of eight weeks, an UC-patient in an active disease status caused outpatient costs of 47.21 Euro from the perspective of the SHI in Germany. We assumed that no outpatient costs were caused by patients in the remission, post-surgery, inpatient or surgery model state.

#### Inpatient costs

In Germany, inpatient stays are reimbursed via lump sums (diagnosis related groups, DRG) as well. The DRGs which were chosen for this analysis were obtained from the German cost study [21] and fitted to 2009. Hence, an inpatient stay without surgery was reimbursed with 1,584.27 Euro, an inpatient stay including surgery with 3,812.55 Euro from the perspective of German SHI (again also taking co-payments by patients into account) [24].

### Analysis

The outputs of the model are expressed as an incremental cost effectiveness ratio (ICER), presenting incremental costs per additional quality adjusted life year (QALY) gained. All costs and QALYs are discounted at an annual rate of 5% according to the current German Recommendations on Health Economic Evaluation (Hannover Consensus) [25]. Uncertainty in the model was assessed using one-way and probabilistic sensitivity analysis (PSA). The PSA was performed with 10,000 Monte Carlo simulations, drawing random values for health state costs, utilities and remission rates, with the observed parameter distribution in the data source.

## Results

On the basis of this deterministic approach (using the mean parameter values) overall healthcare costs for a patient receiving Mezavant were 624 Euro lower than for a patient receiving Asacol over a 5-year time period. Additionally, patients receiving Mezavant gained slightly more QALYs (0.011) in comparison to patients who received Asacol. The results lead to a negative ICER which means that Mezavant is dominant in comparison to Asacol due to lower costs and also higher benefits in terms of QALYs. The results from the probabilistic sensitivity analysis confirm the dominance of Mezavant in comparison to Asacol. Detailed results are shown in table 3.

**Table 3:** Summary of the base case analysis

	Intervention	Estimated 5-year costs	QALY gained	Difference (cost)	Difference (QALY)	Cost per QALY
deterministic	Mezavant	4,940 €	3.320	-624 €	0.011	NA
	Asacol	5,564 €	3.309			
probabilistic	Mezavant	4,939 €	3.320	-705 €	0.022	NA
	Asacol	5,644 €	3.330			

The primary outcome measure of this cost effectiveness analysis was the benefit in terms of a QALY. However, in addition, other measures of effectiveness were also evaluated. Results were consistent with all other analyses. For instance, patients receiving Mezavant spent 18 more days in remission in comparison to Asacol and had 0.03 inpatient episodes as well as 0.002 surgeries less in the time frame of five years as set in the base case of the analysis.

The analysis was also repeated with a lifelong time horizon. According to the calculations, a treatment with Mezavant leads to 1,992 Euro less in costs than Asacol per patient. Furthermore, patients incrementally gain 0.028 QALYs in comparison to Asacol. In total, the lifelong model also results in a dominant alternative with Mezavant.

One-way sensitivity analyses suggest that these findings are driven by differences in the acquisition cost of mesalazine formulations as well as the efficacy of the respective treatments. However, the different long-term remission rates presented by Prantera [14] do not have a major impact on the results. Even if the same long-term efficacy was applied for both treatment arms (e.g. 68%), Mezavant remains dominant over Asacol.

**Figure 2:**
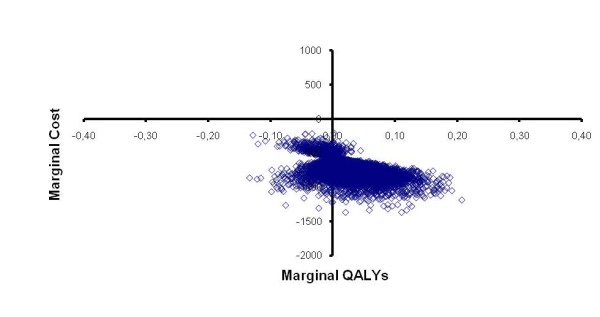
**Scatter plot of PSA analysis**. QALY: quality adjusted life year.

The PSA indicates a probability of 76% for cost savings and higher QALYs with Mezavant compared with Asacol. A scatter plot of the analysis is illustrated in Figure 2.

## Discussion

This study has some limitations which will be addressed in the following.

Within this study, we extrapolated 16-weeks-clinical trial data to a five year time horizon which is associated with some uncertainties. Furthermore, the study from Prantera et al. [14], which was used for the long-term remission maintenance rates, only lasts for one year. However, to our knowledge, no other data exists, which gives information about the long-term effects of Mezavant versus Asacol treatment. Initial remission rates of Mezavant and Asacol were taken from a single study [9] to ensure internal validity (e.g. consistent endpoints or homogeneity of included patients). Nevertheless, other clinical trials (Schroeder et al. [26]; Sninsky et al. [27]) are documented which also investigated the efficacy of Asacol. However, the reported remission rates are in line with the findings of the study of Kamm et al. [9], although other clinical endpoints were set and a six-week-period was chosen as a trial length. All in all, these studies support the conservative assumption included in the present study regarding the inclusion of a remission rate of 61.0% for Asacol-patients receiving an increased dose of mesalazine.

It also might have been reasonable from the clinical trial data to assume an even lower remission rate due to the fact that patients who initially received Asacol and did not achieve a remission in the first eight weeks were henceforth treated with an increased dose of Mezavant. Since the remission rate of Mezavant in the first eight weeks was higher than the rate of Asacol (40.5% versus 32.6%), it could have been also assumed that an increased dosage of Asacol would not have had the same positive effect as an increased dose of Mezavant. Hence, an alteration of the model inputs reflecting the other clinical studies for Asacol might have been even more favorable to Mezavant.

Within the evaluation, it was assumed that patients are fully adherent. Higher adherence is associated with a reduced probability of relapses as many published findings suggest [28,29]. One aspect which influences adherence is the frequency of dosing. For instance, Kane et al. [28] reports adherence rates of maintenance therapy in UC-patients by dosing regimen: 39% for twice daily, 27% for three times daily and 6% for four times daily. Furthermore, the authors examined the relationship between adherence and treatment efficacy and found that adherent patients had an 89% chance of maintaining remission in comparison to patients who were non-adherent (39%). With respect to the two medications in this study, Mezavant is dosed once daily whereas Asacol is dosed three times daily for acute treatment and two times daily for maintenance. Therefore, it might be assumed that the difference between the remission rates of Mezavant and Asacol might have been even higher if adherence were considered within the model.

In addition, it is indicated that UC-patients have an increased risk of developing colorectal cancer (CRC). This risk, however, may be reduced by the adherent use of 5-ASA [30-32]. Therefore, the inclusion of the increased risk of CRC due to non-adherence might also have as well increased the cost effectiveness of Mezavant in comparison to Asacol.

Further uncertainty is connected to the inclusion of surgery associated mortality data from the UK, which medical system is not directly comparable to the German system. However, due to the lack of sufficient German data, it seemed reasonable to include data from another developed European country. Furthermore, the percentage of patients in hospital who needed to undergo a surgery was taken from the German Federal Statistical Office (10.9%). However, this figure refers to the overall risk of surgery in hospital for all UC-patients and is not specific for the UC-population we are focusing on. Therefore, we have checked the importance of this figure by conducting a one-way sensitivity analysis (variation: 5-30%). The results change only slightly (incremental costs over a 5-year-period +/- 20 Euro). This shows that this specific figure has not a great impact on the results of this model.

Further uncertainties are connected to the structure of the model. Treatment for frequently relapsing patients can include oral immunosuppressants or TNF-alpha-inhibiting agents which were not included in the model. However, TNF do not play an important role in the treatment of UC-patients in Germany yet [21]; furthermore, there is no valid data with respect to transition probabilities which could have been included in the model. The inclusion of TNF therapy would probably not have an effect on the original aim of the study (comparison of Asacol and Mezavant) because the population receiving such therapy is very small and both drugs were given the same treatment pathways in the model. Further uncertainty is connected with assumption that patients who suffer a severe relapse/mesalazine failure need hospitalization. On the other hand, some patients may also require an immediate surgery due to a severe relapse. However, no valid data exists for a more precise approach and the model structure includes no surgical or post-surgical complications. Nevertheless, post-surgical complications could likely occur in patients with a long active disease history. Due to lack of robust data with respect to further health states and transition probabilities, the model was not designed to assess post-surgical complications. Thus, only robust and conservative transition probabilities were included into the model calculations.

The resource use and costs which were included in the model are also connected with some uncertainties. The data source used for collection of resource usage and costs [21] is not set as a longitudinal collection of data but on a cross-sectional study design and therefore represents rather a snapshot at a specific time point. However, the study is the most eligible one in the German context as it includes inpatient as well as outpatient resource use, is up to date (2007) and addresses the perspective of the SHI. According to the current German guidelines [22] the drug prices of Mezavant and Asacol were taken from July 2009. To check whether the prices have changed after July 2009 or at the beginning of 2010 (and thereby the relative results of the analysis), the SHI-drug prices were re-calculated using current drug price. The last price change occurred in April 2010. After subtracting discounts and co-payments by patients, the drugs price of Asacol is currently 0.39 Euro per 400 mg and 1.00 Euro per 1,200 mg of Mezavant [23]. Hence, Mezavant still is less costly for the SHI than Asacol. If these new prices were put into the model, the results would have even been more favorable to Mezavant. In general, due to the specialty of the reimbursement system in Germany, the results of this cost-effectiveness-model are not directly transferable to other foreign settings.

There is an urgent need for more research regarding the health care of patients suffering from UC. As stated above, long-term data which gives information on the long-term effects of specific UC-therapies are missing to date. Therefore, in models efficacy data from clinical trials needs to be extrapolated which is associated with uncertainties. Furthermore, utility data for UC-patients is very rare. The only utilities which were eligible for this cost effectiveness model came from two studies that were only available in an abstract format [19,20]. Therefore, there is an urgent need to collect utility data for patients with UC, also for different country settings.

To our knowledge, there exists no cost effectiveness model for the German setting yet, which compares mesalazines in the treatment of UC. Hence, it is not possible to compare the results of this study with other German publications. This indicates a need for further health economic research in this area to identify cost effective options in the treatment of UC-patients in Germany.

In summary, the analysis shows that over a five year as well as a lifelong time horizon, Mezavant is less expensive and results in higher gains of QALYs compared with Asacol. The analysis and its results are reasonably robust as the PSA shows.

## Conclusions

The analysis suggests that patients treated with Mezavant are in remission longer, gain more QALYs, and incur lower direct costs to the SHI compared with patients receiving Asacol. Mezavant may therefore be a suitable first-line option for the induction and maintenance of remission in UC.

## List of abbreviations

NA: not applicable; PSA: probabilistic sensitivity analysis; QALY: Quality adjusted life years; SHI: Statutory health insurance; UC: ulcerative colitis.

## Competing interests

Financial support provided by Shire Pharmaceuticals Inc. **Remark: **The trademark Asacol^® ^is owned by Tillotts Pharma AG, Zeifen, Switzerland. The trademark Mezavant^® ^is licensed from Giuliani International Ltd., Dublin. Ireland.

## Authors' contributions

AP has gathered the necessary data, run the model as well as analyzed and interpreted the results. She has also written the manuscript. LY has helped to design the model as well as to analyze and interpret the results. TM and JMS have helped to interpret the results as well read the manuscript critically. All authors have read and approved the final manuscript.

## Pre-publication history

The pre-publication history for this paper can be accessed here:

http://www.biomedcentral.com/1472-6963/11/157/prepub
